# Organ Dysfunction in Sepsis-associated Intravascular Coagulation

**DOI:** 10.14789/jmj.JMJ23-0042-P

**Published:** 2024-02-10

**Authors:** MARCEL LEVI, TOSHIAKI IBA

**Affiliations:** 1 Department of Vascular Medicine, Amsterdam University Medical Center, Amsterdam, the Netherlands; 1 Department of Vascular Medicine, Amsterdam University Medical Center, Amsterdam, the Netherlands; 2 Department of Medicine, University College London Hospitals NHS Foundation Trust, and Cardio-Metabolic Programme-NIHR UCLH/UCL BRC London, London, UK; 2 Department of Medicine, University College London Hospitals NHS Foundation Trust, and Cardio-Metabolic Programme-NIHR UCLH/UCL BRC London, London, UK; 3 Department of Emergency and Disaster Medicine, Juntendo University Graduate School of Medicine, Tokyo, Japan; 3 Department of Emergency and Disaster Medicine, Juntendo University Graduate School of Medicine, Tokyo, Japan

**Keywords:** sepsis, disseminated intravascular coagulation, acute kidney injury, thrombus, neutrophil

## Abstract

Sepsis is frequently associated with disseminated intravascular coagulation (DIC) and multiple organ damage. It is widely accepted that DIC is not merely a complication but also plays a role in the development of organ dysfunction. Thrombus formation in the microvasculature leads to impaired tissue perfusion and organ damage. Activated neutrophils interacting with platelets, endothelial injury, and an imbalance of coagulation and fibrinolysis are the essence of thromboinflammation induced in sepsis-associated DIC. The above mechanisms are typically seen in sepsis-associated acute kidney injury (AKI), and the development of AKI is known to be strongly associated with the severity of sepsis. It is important to recognize the pathway of this mechanism in the context of sepsis management.

Disseminated intravascular coagulation (DIC) is defined as “an acquired syndrome characterized by the intravascular activation of coagulation with loss of localization arising from different causes. It can originate from and cause damage to the microvasculature, which if sufficiently severe, can produce organ dysfunction” in 2001^1)^. As time has passed, we now understand that intravascular inflammation and subsequent immunothrombus formation, along with endothelial damage, are the principal mechanisms of organ dysfunction in sepsis-associated DIC, and this process is termed “thromboinflammation”^2)^. Thromboinflammation is a fundamental host response to severe and/or systemic infection and is recognized not only in sepsis but also in various diseases such as trauma, heat stress, and virus infection, including coronavirus disease 2019. Although major players, coagulation/fibrinolytic systems, monocytes, neutrophils, platelets, and vascular endothelial cells, are the same, the detail of the mechanisms differs slightly between the underlying diseases, with immunothrombus-induced tissue malcirculation being the primary mechanism of organ dysfunction in sepsis^3)^.

Among various targets, the kidney is one of the most frequently affected organs in sepsis, but the incidence is significantly different between various reports. White et al.^4)^ performed a retrospective cohort study in 12 intensive care units from 2015 to 2021 and reported that approximately 16% of septic patients met the acute kidney injury (AKI) criteria (13,451 out of 84,528), and about half of them were stage 1. Meanwhile, cases with stage 3 and required renal replacement therapy were only 21% and approximately 3% of total septic patients. However, it is also known that the incidence of severe AKI increases dramatically when DIC is present, and Helms et al.^5)^ reported that 47.3% of patients with sepsis-associated DIC and shock required renal replacement therapy, compared to 21.3% of patients without DIC (*P* < 0.001). Furthermore, the complication of AKI considerably enhances the severity of sepsis. Seymour et al.^6)^ analyzed data from over 60,000 septic patients by using machine learning techniques and subdivided them into four sepsis phenotypes. Among phenotypes, the α phenotype was the most frequent (33%), although less severe and requiring less vasopressor support. Patients in the β phenotype (27%) were older and had more chronic illnesses. Those in the γ phenotype (27%) exhibited more inflammation and pulmonary dysfunction. The δ phenotype (13%) represented the most severe cases with a higher incidence of AKI development, hepatic dysfunction, and endothelial dysfunction, and a greater risk of death at 32% was reported. Of note, the coagulation markers were significantly greater in the δ phenotype compared to the others. These findings suggest a tight connection between DIC and AKI, and their association with poorer outcomes in sepsis.

Regarding the etiology of sepsis-associated AKI, Peerapornratana et al.^7)^ described three major factors: microvascular dysfunction, inflammation, and metabolic reprogramming. In addition to those mechanisms, we think the microclots formed by the neutrophils and platelets (immunothrombus) play a pivotal role. Microclot-mediated tissue malcirculation is a common scenario in other organ dysfunctions^8)^; however, the kidney can be more significantly affected due to its anatomical nature during DIC. The renal tubular cell is generally considered the primary target of injury in sepsis. It is known that tubular cell-specific biomarkers such as neutrophil gelatinase-associated lipocalin (NGAL) and kidney injury molecule 1 (KIM-1) elevate as the increased damage to the kidney^9)^. However, Kidney Disease Improving Global Outcomes (KDIGO) is recognized to be a standard scale for evaluating AKI in sepsis, and the urine output and serum creatinine level, which consists KDIGO scale, were primarily determined by the volume of glomerular filtration. In a rat model of sepsis-associated DIC, we observed that the glomerular capillaries are filled with adhered neutrophils, and the blood flow was significantly reduced (Figure 1). The unique structure of the renal circulation is characterized by the presence of a glomerular capillary between the afferent and efferent arterioles, ultimately supplying the blood flow to the renal tubular system. When microthrombi are formed in the glomerular capillary, not only the glomerular filtration but the renal tubular system is also disturbed ^10, 11)^. AKI in sepsis encompasses two subtypes i.e., glomerular damage-predominant type and tubular cell damage- predominant type, and DIC is more likely to be associated with the former type. We assume dividing AKI into subtypes may help in planning therapeutic strategy.

**Figure 1:**
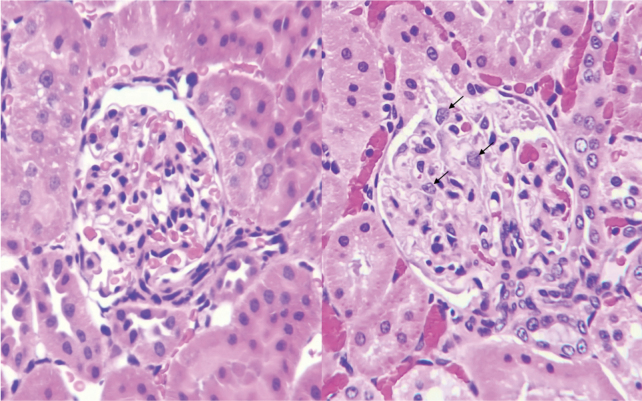
Pathologic findings of the kidney Sepsis-associated disseminated intravascular coagulation (DIC) was induced by intravenous infusion of *E. coli.* Rats were sacrificed 2.5 hours after the infusion, and kidney specimens were stained with hematoxylin and eosin. In the normal kidney (left), red cells and the capillary lumen were observed. In contrast, in the kidney of a septic DIC rat, the glomerular capillary was occupied by leukocytes (arrows), and the capillary lumen was hardly visible (right). Mild renal tubular cell injury was observed in the septic DIC rat.

In summary, AKI is a common complication in sepsis, and severe AKI frequently appears when the patients are complicated by DIC. Since severe AKI is tightly associated with high mortality, it is crucial to understand the pathomechanism. The development of AKI is owing to its unique vascular structure, and immunothrombus plays a major role in the pathogenesis.

## Funding

No funding was received.

## Author contributions

ML and TI wrote and reviewed the manuscript. Both authors read and approved the final manuscript.

## Conflicts of interest statement

The authors declare that they have no conflict of interest.
